# Kenyan Free-Tailed Bats Demonstrate Seasonal Birth Pulse Asynchrony with Implications for Virus Maintenance

**DOI:** 10.1007/s10393-024-01674-x

**Published:** 2024-02-19

**Authors:** Tamika J. Lunn, Reilly T. Jackson, Paul W. Webala, Joseph Ogola, Kristian M. Forbes

**Affiliations:** 1 Department of Biological Sciences, University of Arkansas, Science and Engineering Building, 850 W Dickson St, Fayetteville, AR 72701 USA; 2 Odum School of Ecology, University of Georgia, Athens, GA 30602 USA; 3 Center for the Ecology of Infectious Diseases, University of Georgia, Athens, GA 30602 USA; 4 Wildlife Research Branch, Arizona Game and Fish Department, Phoenix, AZ 85086 USA; 5 Department of Forestry and Wildlife Management, Maasai Mara University, Narok, 20500 Kenya; 6 Department of Medical Microbiology, University of Nairobi, Nairobi, 19676 Kenya

**Keywords:** Africa, Chaerephon, Ebola virus disease, Filovirus, Molossidae, Transmission

## Abstract

Ecological information on wildlife reservoirs is fundamental for research targeting prevention of zoonotic infectious disease, yet basic information is lacking for many species in global hotspots of disease emergence. We provide the first estimates of synchronicity, magnitude, and timing of seasonal birthing in *Mops condylurus*, a putative ebolavirus host, and a co-roosting species, *Mops pumilus* (formerly *Chaerephon pumilus*). We show that population-level synchronicity of *M. condylurus* birthing is wide (~ 8.5 weeks) and even wider in *M. pumilus* (> 11 weeks). This is predicted to promote the likelihood of filovirus persistence under conditions of bi-annual birthing (two births per year). Ecological features underlying the magnitude of the birth pulse—relative female abundance (higher than expected for *M. condylurus* and lower for *M. pumilus*, based on literature) and reproductive rate (lower than expected)—will have countering effects on birthing magnitude. Species-specific models are needed to interpret how identified birth pulse attributes may interact with other features of molossid ebolavirus ecology to influence infection dynamics. As a common feature of wildlife species, and a key driver of infection dynamics, detailed information on seasonal birthing will be fundamental for future research on these species and will be informative for bat-borne zoonoses generally.

**Supplementary Information:**

The online version contains supplementary material available at 10.1007/s10393-024-01674-x.

## Introduction and Purpose

Growing concern about the emergence of zoonotic infectious diseases has stimulated research into the dynamics and drivers of infection in wildlife reservoir hosts (Patz et al. 2004; Jones et al. 2008). Understanding factors that drive the magnitude and timing of epidemics—and which allow for the long-term persistence of pathogens—in animal host populations is of particular importance to the prediction and prevention of zoonotic spillover into humans (Plowright and Hudson 2021). Investigations into how ecological features shape infection is achieved through empirical and theoretical research at the interface of ecology and wildlife epidemiology (e.g., Peel et al. 2014; Lunn et al. 2021; Mancy et al. 2022). The availability of key ecological information on wildlife reservoir hosts is fundamental to these research efforts, yet even basic information is lacking for many wildlife species in global hotspots of infectious disease emergence (Kuzmin et al. 2011).

Seasonal birthing (i.e., the synchronized, seasonal birth of juveniles during a “birth pulse”) is a common ecological feature of wildlife species and is thought to play a key role in the infection dynamics of populations via the addition of susceptible individuals (Peel et al. 2014; Plowright et al. 2016; Baker et al. 2020). At its simplest, the seasonal addition of naïve juveniles increases the proportion of contacts between susceptible and infected individuals, increasing the rate of transmission during and following the birthing period, thereby driving seasonal epidemic cycles (Peel et al. 2014). Ecological attributes of seasonal birthing, as well as other host and pathogen features such as population size and infectious period, shape variation in this dynamic host–pathogen relationship.

Synchronicity of birthing (the time period over which birthing occurs), the number of birth pulses per year, and the magnitude (number of births) in each pulse are key drivers of epidemic cycles and contribute to the probability of pathogen extinction from a population (Peel et al. 2014; Hayman 2015). More synchronous birth pulses (births over a shorter time) can drive pathogen extinction by creating large amplitude oscillations in epidemics (i.e., high peaks, low troughs), which increase the probability of stochastic fade-out during troughs in the epidemic cycle (Peel et al. 2014). This is exacerbated by short infectious periods and high demographic turnover and increases the population size required for pathogen persistence (Peel et al. 2014; Hayman 2015). Meanwhile, having more birth pulses per year can increase the likelihood of pathogen persistence by decreasing the amplitude of oscillations (i.e., lower peaks, higher troughs), meaning the number of infected individuals does not drop as low between birth pulses, and the risk of pathogen extinction via stochastic fade-out is reduced (Hayman 2015). The number of births per pulse impacts the influx of susceptible juveniles into the population and may tune the effects from synchronicity and frequency of birth pulses (e.g., Amman et al. 2012).

Modeling has shown that bi-annual birth pulses (two birth pulses per year) may be needed for the persistence of filoviruses (including *Ebolavirus* sp. and *Marburgvirus* sp.) in bat populations, and that persistence—even with bi-annual birthing—is sensitive to birth synchrony and host/virus attributes (Hayman 2015). Persistence of filovirus is more likely when birth synchronicity is low, when the incubation or infectious periods are long, and when population sizes are large (Hayman 2015). These findings highlight ecological conditions under which sustained transmission of Ebola and Marburgviruses may be possible and identify key traits of reservoir hosts. However, these results pertain to generic bat populations with fruit bat-like ecological traits (based on the African fruit bats, *Eidolon helvum* and *Rousettus aegyptiacus*) and are not necessarily applicable to other possible hosts of ebolaviruses, including insectivorous bats (Hayman 2015; Leendertz et al. 2016). Species-specific investigations into plausible reservoir hosts, especially for ebolaviruses, remain limited by deficits in information on bat ecology. The synchronicity of birthing—in particular—is unknown for most bat species and warrants further attention (Kuzmin et al. 2011; Hayman 2015).

The Angolan free-tailed bat (Molossidae: *M. condylurus*) has been implicated as a reservoir host for ebolaviruses. Wild-caught *M. condylurus* have tested positive for ebolavirus antibodies (De Nys et al. 2018) and demonstrate the ability to replicate and shed ebolaviruses following experimental inoculation, with little evidence of virus-induced morbidity (Swanepoel et al. 1996; Edenborough et al. 2019). More recently, this species has been associated with a new ebolavirus, Bombali virus, with detection of viral RNA in wild-caught bats from five distinct locations across their range (Sierra Leone, Guinea, two locations in Kenya, and Mozambique) (Goldstein et al. 2018; Forbes et al. 2019; Karan et al. 2019; Kareinen et al. 2020; Lebarbenchon et al. 2022). Bombali virus RNA has also been detected from a closely related co-roosting species, *M. pumilus* (formerly *Chaerephon pumilus*) (Goldstein et al. 2018)*.*

Birth pulse synchronicity and magnitude have not been estimated for *M. condylurus* or *M. pumilus*, despite the pertinence of this information to understanding infection dynamics. The temporal resolution of existing research—at best, singular capture events once per month—is insufficient to accurately estimate the spans of most reproductive states in bats (Mutere 1973; O’Shea and Vaughan 1980; Happold and Happold 1989; Vivier and Van Der Merwe 1996; Vivier and Merwe 1997). Gestation and lactation periods have been estimated from patchy data, at roughly 85 and 50–60 days, respectively, for *M. condylurus*, and 67–72 and 21–28 days for *M. pumilus* (McWilliam 1976; Happold and Happold 1989; Vivier and Merwe 1997). The timing and frequency of birth pulses vary by latitude, though female *M. condylurus* show bi-annual birthing across their range, and *M. pumilus* up to penta-annual birthing (five birth pulses per year) (McWilliam 1976; Happold and Happold 1989). The proportion of females achieving the maximum of five births per year has not determined, but is likely a rare occurrence (McWilliam 1976). Both species have a post-partum oestrus, where bats can be detected lactating and gestating simultaneously (Happold and Happold 1989).

Given current gaps in knowledge, and the importance of detailed reproductive information for investigating viral maintenance, the purpose of this study was to empirically characterize key parameters of the birth pulse in a population of *M. condylurus* and *M. pumilus* bats. Specifically, we investigated the timing, synchronicity, and magnitude of their respective birth pulses in south-eastern Kenya, which is a global hotspot for disease emergence (Jones et al. 2008), and a location where Bombali virus has been detected (Forbes et al. 2019; Kareinen et al. 2020). We contextualize our results with prior research on female reproduction for these species.

## Methods

**Figure 1:**
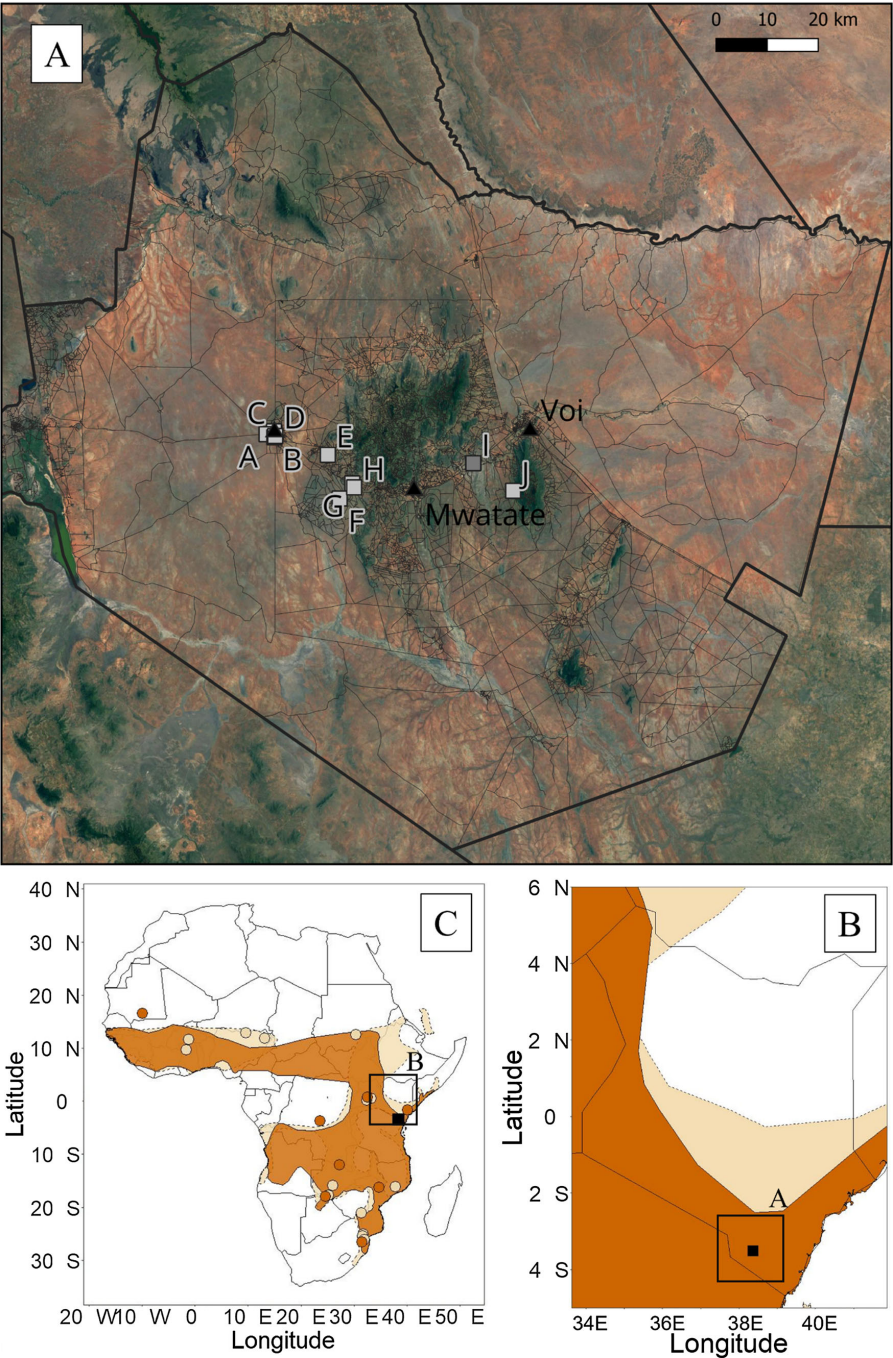
Study sites in the Taita-Taveta county (panel A). Building roosts are shown as light gray squares (sites A-H and J) and bridge roosts as dark gray squares (site I). The distributions of *M. condylurus* (brown) and *M. pumilus* (beige) are shown throughout Kenya (panel B) and Africa (panel C), along with the location of the study area (black square) and previous studies on female reproduction for these species (as described in Tables 1 and 2, locations approximate). Species distribution data is from IUCN (2021) (Color figure online).

We captured bats over a 12-week period between January 30th and April 14th, 2022, timed to encompass the dry season (January to February) and the beginning of the wet season (March to May) in Taita-Taveta County, Kenya (Tuure et al. 2020). This is the predicted timing of parturition for these species at this location (pers. comm. by T. J. O'Shea cited in Happold and Happold (1989)). We targeted *M. condylurus* and *M. pumilus* bats by netting at building and bridge roosts thought or known to contain these species (Fig. 1). Bats were captured 1–4 times per week from 10 roost sites (Appendix S1) using mist nets (Ecotone 716/3-12P) set at or above roosting height (for buildings and bridges) and handheld butterfly nets (Hyönteistarvike Tibiale Oy, 65 cm net and 550 cm telescopic pole) to block additional, smaller entrance/exit points of buildings not covered by mist nets (buildings only). Nets were either opened at dusk to capture bats leaving the roost to forage or opened from approximately one hour after dusk and operated through the night (17:30–22:30), to capture bats returning to the roost after foraging. Bats were removed from the nets immediately to minimize stress or injury. Where feasible, bats were also captured directly from roosting spaces during daytime hours by entering the roost space and removing as many as possible. Bats were held in individual cloth bags and sampled the morning following capture (for night netting), or the same day (in the case of daytime roost capture).

Demographic information (sex, age, and reproductive status) and standard measurements (weight and forearm length) were recorded for all captured bats. Age (juvenile or adult) was determined by evidence of current or previous reproduction, size, and ossification of the epiphyses (juveniles having an incomplete ossification, and adults having a complete ossification) (Anthony 1988; Monadjem et al. 2007). We categorized reproductive status into five states for females: non-reproductive, pregnant and not lactating, pregnant and lactating, lactating and not pregnant, and post-lactating (Happold and Happold 1989). Nipples that were lactating were discernible by a bare patch of skin around an engorged nipple (Linton and Macdonald 2018). Post-lactating nipples were those that appeared visibly engorged but did not have a bare patch of skin (i.e., not presently lactating but which might have lactated recently) (Linton and Macdonald 2018). Gestation was noted by gentle palpation of the abdomen. If a uterine bulge was present (not palpable on juveniles, subadults or males), pregnancy was classified as early (the uterine bulge was palpable but not significant), mid (the fetus was able to be felt, but the females’ abdomen did not extend past the ribs), or late (the fetus/bulge was obviously wider than the female’s rib cage). Note that bats were processed in the morning so that their stomachs were not full of food at the time of processing. The stomach distention (observed exclusively in adult females) was deemed to be pregnancy and not food. Females classified as non-reproductive were not detectably pregnant, lactating or recently lactating (i.e., without enlarged nipples) (Happold and Happold 1989). Species were identified following Patterson and Webala (2012). Note that roosts comprise mixed sexes, mixed ages, and mixed reproductive statuses; segregation according to age, sex, and reproductive status does not occur at any time of year for these species (Marshall and Corbet 1959; Mutere 1973; Happold and Happold 1989).

Individual *M. condylurus* and *M. pumilus* bats of appropriate size (with a forearm length between roughly 31–54 mm) were banded (Porzana, Ltd., Icklesham, East Sussex, UK) with numbered, 2.9 mm, aluminum alloy, split-lipped rings to allow identification upon recapture. Post-processing, bats were released at the roost after dusk. Bats were processed, as described above, on each capture or recapture. Personal protective equipment was worn at all times during capture, sampling, and release of bats. Procedures involving bat captures and handling followed established guidelines (Sikes et al. 2016) and animal ethics approvals (see “Ethical approval” at end of manuscript).

We define birth pulse synchronicity as the time period over which birthing occurs, for which we consider ‘the beginning’ as the first observation of females lactating but non-gestating (indicative of recent birth), and ‘the end’ as the last observation of gestating females. As these observations may not reflect the broader population (i.e., may show individual extremes), we also consider gestational stage per week—if all females at a single time point are observed to be at the same stage of gestation, this would indicate that females are highly synchronous in mating and birthing. In contrast, if females at a single time point are observed to be a mix of early-, mid- and late-stage gestation, this would indicate that mating and birthing are not so highly synchronous. To this end, we used Chi-square tests to evaluate birth pulse synchronicity in this population of *M. condylurus* and *M. pumilus* bats. Comparisons were made per week and included: (1) numbers of gestating vs non-gestating females; (2) numbers of females at early vs mid- vs and late gestational stages; and (3) numbers of females that were lactating and gestating simultaneously, vs those lactating but not gestating, vs those that were post-lactating. We excluded recaptures of individuals captured < 1 week apart, to ensure there was no repetition of individuals within single Chi-square tests. Recaptured bats were included across independent Chi-square tests (e.g., a female caught in weeks one and ten would be included in the Chi-square test of week one, and the Chi-square test of week ten; both independent tests). All analyses were repeated with data excluding all recaptures to check the influence of recaptures throughout the tests (Appendix S2). We also performed a Chi-square test on the overall dataset of individuals (i.e., no recaptures included) to compare the number of males and females in the population, as relative female abundance is an important feature underlying the magnitude of the birth pulse. All analyses were undertaken in R version 4.2.2 (R Core Team 2022) using the ‘stats’ (v4.2.2) package.

## Results

We captured 391 individual *M. condylurus* and 472 individual *M. pumilus* across the study period. The demographic breakdown of bats is given in Appendix S3. The male-to-female ratio of the population was nearly 1:1 for *M. condylurus* (1:1.08, 188 individual males, vs 203 individual females), and nearly 1:2 for *M. pumilus* (1:1.84, 166 individual males, vs 306 individual females). *M. pumilus* sex ratio deviated significantly from equal (χ^2^ = 41.5, *p* < 0.0001, vs χ^2^ = 0.6, *p* = 0.4815 for *M. condylurus*). The remainder of the results presented pertain to adult females of the two species, as the scope of the manuscript is on the female birth pulse.

We captured 175 adult, female *M. condylurus* individuals, and 278 adult, female *M. pumilus* individuals. Recapture of adult females was low for both species, with 4.6% of *M. condylurus* (8 of 175 individuals) and 4.7% of *M. pumilus* (13 of 278 individuals) recaptured more than once. Data on recaptures are provided in Appendix S4. With recapture events included, a total of 185 adult female *M. condylurus* and 291 adult female *M. pumilus* were captured.

**Figure 2:**
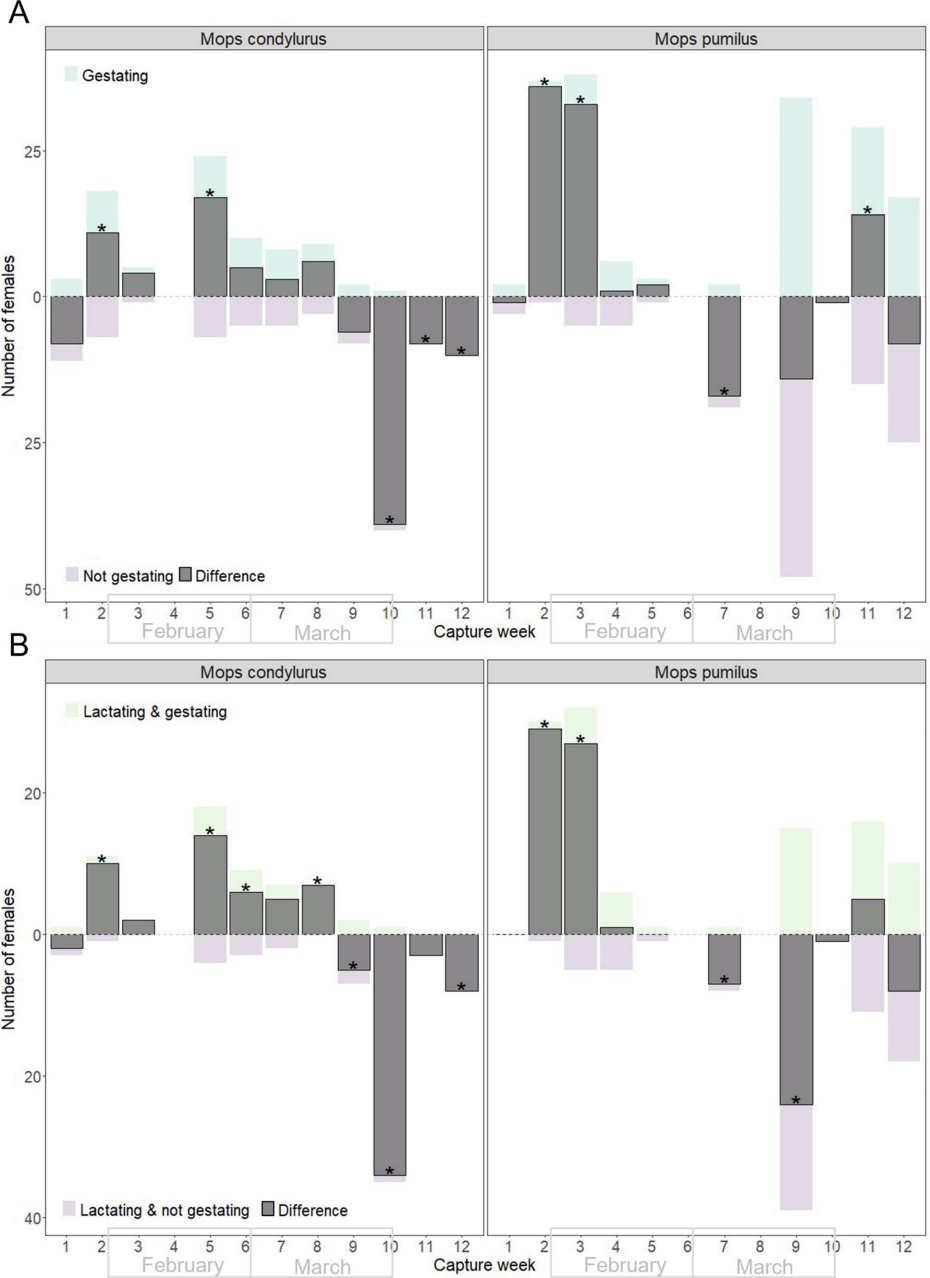
Frequency of reproduction categories observed across the 12-week period. Panel A shows the number of gestating (teal, above 0) and non-gestating (purple, below 0) females. Panel B shows the number of females lactating and gestating (green, above 0), and lactating but not gestating (purple, below 0). In both panels, the difference between the two groups is overlaid (gray), and weeks with significantly different numbers of each group are marked with an *. Weeks span the last week in January to the second week of April 2022. There is no repetition of individuals within weeks (within single Chi-square tests), but there is repetition of individuals across weeks (across independent tests) (Color figure online).

Seventy-seven individual *M. condylurus* and 165 individual *M. pumilus* were gestating. Three individuals of each species were recaptured within the gestational period (at different weeks), yielding 80 and 168 total captures of gestating females for each species, respectively (Appendix S3). Significantly more gestating than non-gestating *M. condylurus* were captured at the beginning of the pulse (week 2: χ^2^ = 4.84, *p* = 0.0420; and week 5: χ^2^ = 9.32, *p* = 0.0038) (Fig. 2A). Through the middle of pulse (weeks 6–9), the number of gestating and non-gestating *M. condylurus* were comparable. By the end of the pulse (weeks 10–12), significantly more *M. condylurus* were not gestating than gestating (week 10: χ^2^ = 37.10, *p* = 0.0001, week 11: χ^2^ = 8.00, *p* = 0.0084, week 12: χ^2^ = 10.00, *p* = 0.0019, Fig. 2A). This supports a pattern of seasonal birthing in this species. There was a less consistent pattern for gestating versus non-gestating *M. pumilus*—there were significantly more gestating females at both the beginning and end of the study (week 2: χ^2^ = 34.11, *p* = 0.0001; week 3: χ^2^ = 25.33, *p* = 0.0001; and week 11: χ^2^ = 4.46, *p* = 0.0487) and significantly more non-gestating females in the middle (week 7: χ^2^ = 13.76, *p* = 0.0002), while numbers of gestating and non-gestating females did not differ at intermittent weeks throughout (weeks 4, 5, 9, 10, and 12) (Fig. 2A). This may indicate that birthing is less seasonal for this species at this location.

**Figure 3:**
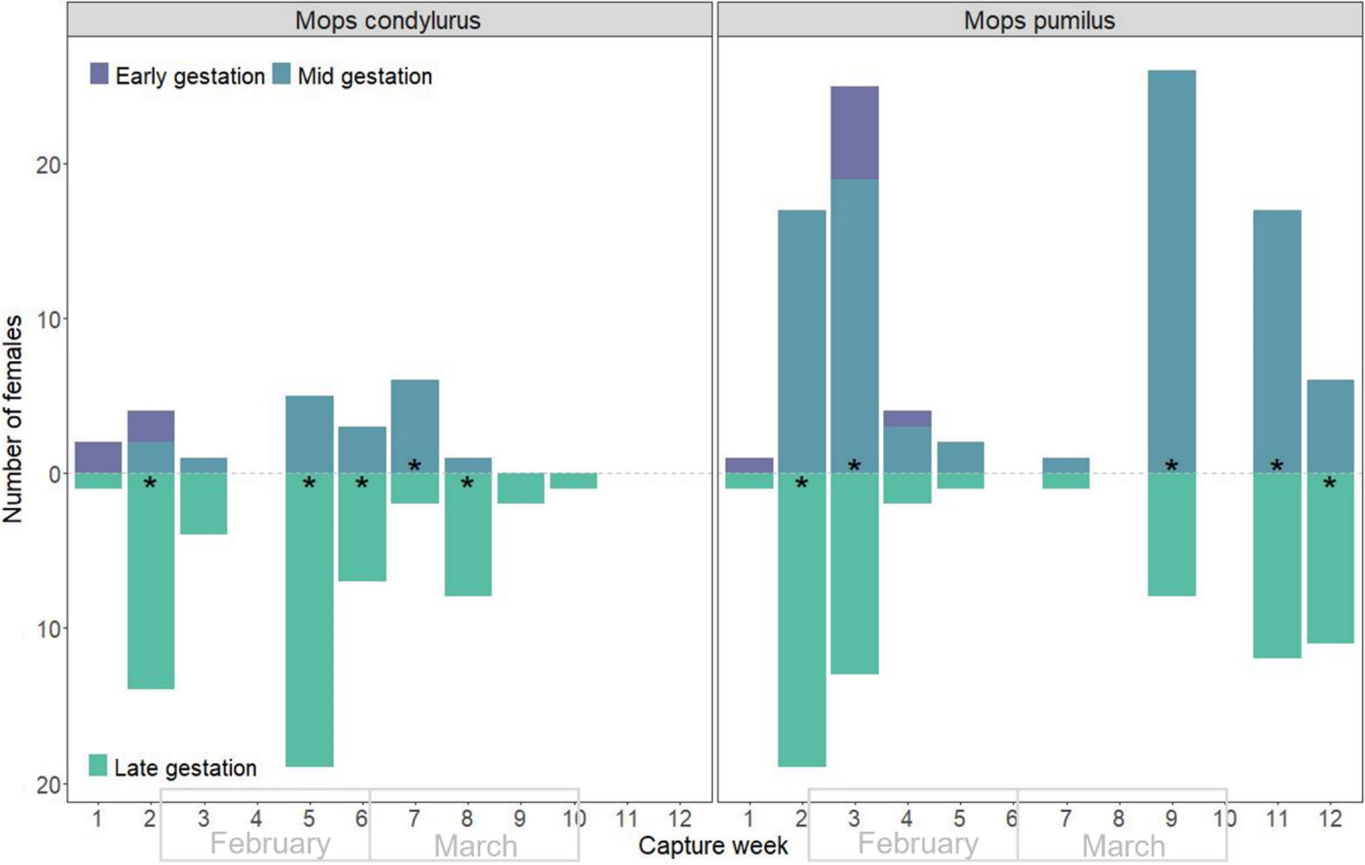
Frequency of gestation stages observed across the 12-week period. Early- and mid-stage females are stacked (above 0). Weeks with significantly different numbers of each group are marked with an *. Weeks span the last week in January to the second week of April 2022. There is no repetition of individuals within weeks (within single Chi-square tests), but there is repetition of individuals across weeks (across independent tests) (Color figure online).

Stage of gestation was not completely synchronous within the population, though tracked the progression of a birth pulse for *M. condylurus*. For this species, females of all gestational stages (early, medium, and late stages) were detected at the beginning of the pulse (weeks 1–2). Females at medium- and late-stage gestation were detected through the middle of the pulse (weeks 3–8), and at late-stage gestation only, by the end of the pulse (weeks 9–10) (Fig. 3). The co-occurrence of disparate gestational stages within weekly timepoints suggests a level of asynchrony in mating and timing of birth for this species. However, late-stage gestation was the most commonly observed gestational stage throughout the study period (significantly more females at late-stage gestation in week 2: χ^2^ = 16.00, *p* = 0.0005; week 5: χ^2^ = 24.25, *p* = 0.0001; week 6: χ^2^ = 7.40, *p* = 0.0222; and week 8: χ^2^ = 12.67, *p* = 0.0028; but see significantly less at week 7: χ^2^ = 7.00, *p* = 0.0341) (Fig. 3). Given their ~ 85-day gestation time (Vivier and Merwe 1997) this suggests the majority of females mate well ahead of the birth pulse. For *M. pumilus,* there was a longer overlap in highly disparate gestational stages, with early-stage gestations detected throughout the first four weeks, and mid- and late-stage gestations detected throughout the entire study period (weeks 2–12, and weeks 1–12, for mid- and late stages, respectively). Both mid- and late stages of gestation were common through the study period which, given similar gestational time (67–72 days, Happold and Happold (1989)) may indicate less synchrony in mating ahead of the birth pulse (week 2: χ^2^ = 18.17, *p* = 0.0001; week 3: χ^2^ = 6.684, *p* = 0.0359; week 9: χ^2^ = 31.29, *p* = 0.0001; week 11: χ^2^ = 15.79, *p* = 0.0006; week 12: χ^2^ = 10.71, *p* = 0.0067, Fig. 3).

In total, 116 and 191 lactating *M. condylurus* and *M. pumilus* individuals were captured. Six *M. condylurus* individuals were lactating at multiple points (five captured twice each, one captured four times, over separate weeks), and nine *M. pumilus* (all captured twice each over separate weeks), yielding 124 and 200 total captures of lactating females for each species, respectively (Appendix S3). Captures of lactating bats spanned nearly the entire study period for both species (weeks 1–12 and 2–12, respectively) (Fig. 2B). Of these, 55 and 109 individuals of each species were gestating while lactating. All stages of gestation (early, mid, and late) were observed in lactating bats, for both species (Appendix S3). Females that were simultaneously lactating and gestating were most often caught between weeks 2–8 for *M. condylurus* (with significantly more observed for weeks 2, 5, 6, and 8, specifically) (week 2: χ^2^ = 11.20, *p* = 0.0032; week 5: χ^2^ = 16.88, *p* = 0.0005; week 6: χ^2^ = 10.50, *p* = 0.0035; week 8: χ^2^ = 14.00, *p* = 0.0013). This swapped to females lactating and not gestating at week 9, with significantly more non-gestating lactating bats observed in weeks 9, 10, and 12 (week 9: χ^2^ = 8.67, *p* = 0.0165; week 10: χ^2^ = 66.17, *p* = 0.0001; week 12: χ^2^ = 12.67, *p* = 0.0025) (Fig. 2B). This timepoint may mark the peak then decline of the birth pulse. For *M. pumilus*, there was a similar though less consistent pattern, with significantly more females lactating and gestating at weeks 2 (χ^2^ = 49.27, *p* = 0.0001), and 3 (χ^2^ = 34.71, *p* = 0.0001), then more non-gestating lactating females at weeks 7 (χ^2^ = 9.80, *p* = 0.0123) and 9 (χ^2^ = 27.28, *p* = 0.0001) (Fig. 2B). For all aforementioned comparisons, there were no substantial differences between results that included or excluded recaptured bats (Appendix S2).

**Figure 4:**
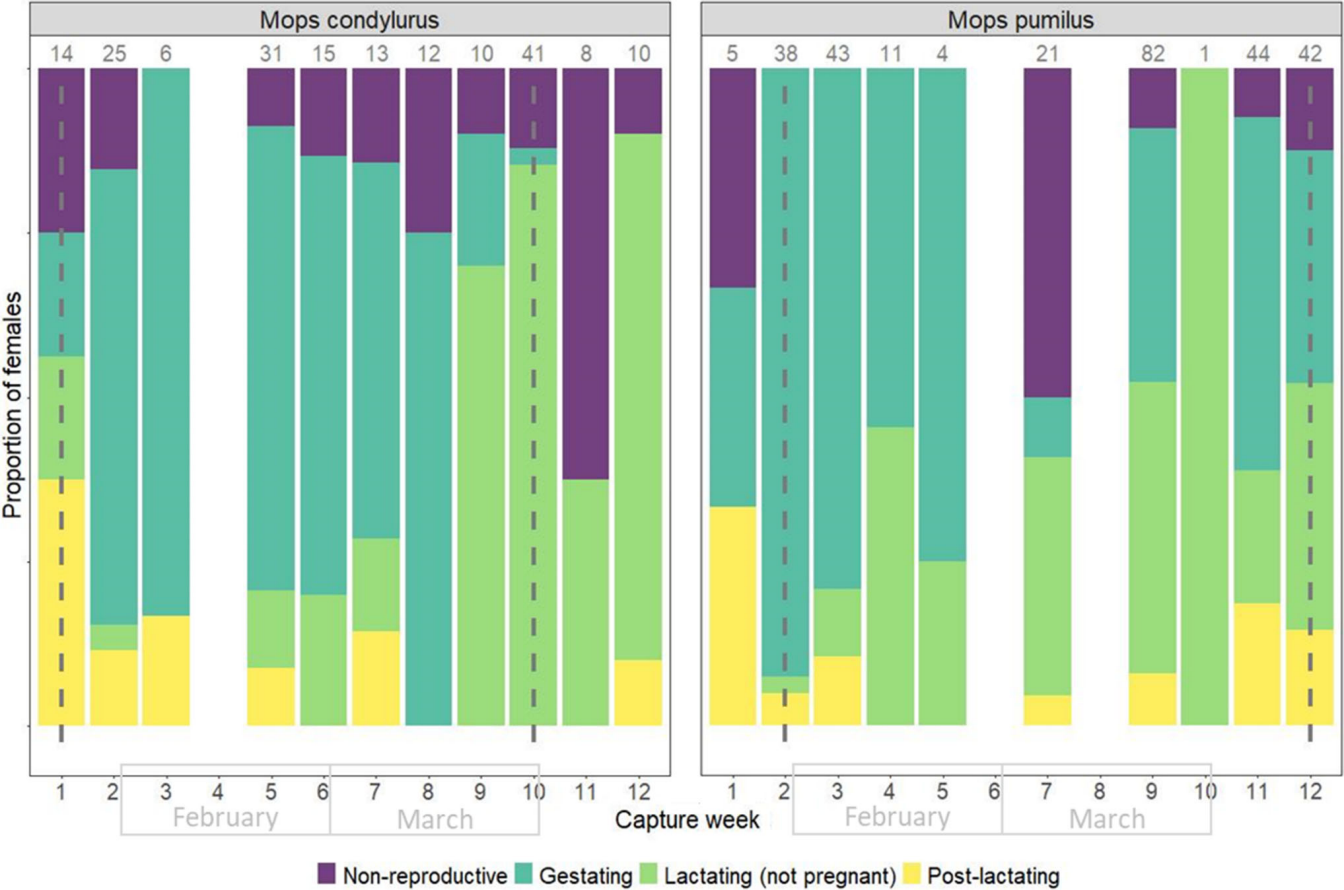
Breakdown of female *M. condylurus* and *M. pumilus* reproductive states across a single birth pulse in south-eastern Kenya. We define birth pulse synchronicity as the time period over which birthing occurs, for which we consider ‘the beginning’ as the first observation of females lactating but non-gestating (indicative of recent birth, left dashed line), and ‘the end’ as the last observation of gestating females (right dashed line). Bars show the relative frequency of bats captured at each reproductive state. Weeks span the last week in January to the second week of April 2022. The total number of adult, female bats caught per week are shown above bars. There is no repetition of individuals within weeks (within single Chi-square tests), but there is repetition of individuals across weeks (across independent tests) (Color figure online).

The duration between the first lactating non-pregnant *M. condylurus* (potentially indicative of the first parturition and start of the birth pulse) and the last gestating *M. condylurus* (indicative of the last parturition and end of the birth pulse) was ~ 8.5 weeks in our study (30th January—31st March) (Fig. 4). For *M. pumilus*, gestating females were captured continuously throughout the study period, up to and including the last week of capture, week 12 (14th April).

Non-reproductive adult females were caught over the entire duration of the study, for both species (Appendix S3). This included 30 captures of 29 individual *M. condylurus*, and 31 captures of 29 individual *M. pumilus*. To be conservative in this estimate, non-gravid females with engorged but non lactating nipples (i.e., post-lactating females) were not classified as non-reproductive, even though they may have reproduced in the previous birth pulse only.

## Discussion

We characterize the reproductive phenology of two free-tailed bat species in Kenya—*M. condylurus* and *M. pumilus*—and present quantitative information on the synchronicity, magnitude, and timing of the birth pulses in these populations. Our data are the highest resolution available for these species across their distributions (see literature in Tables 1 and 2). This allowed us to empirically estimate population birth pulse synchrony for the first time in *M. condylurus* and *M. pumilus*, providing ecological information relevant for understanding mechanisms of transmission and maintenance for ebolaviruses and other zoonotic pathogens in bat populations (Peel et al. 2014; Hayman 2015). As building-roosting bat species, this information will be particularly pertinent for predicting pathogen exposure risk to human inhabitants.

**Table 1:**
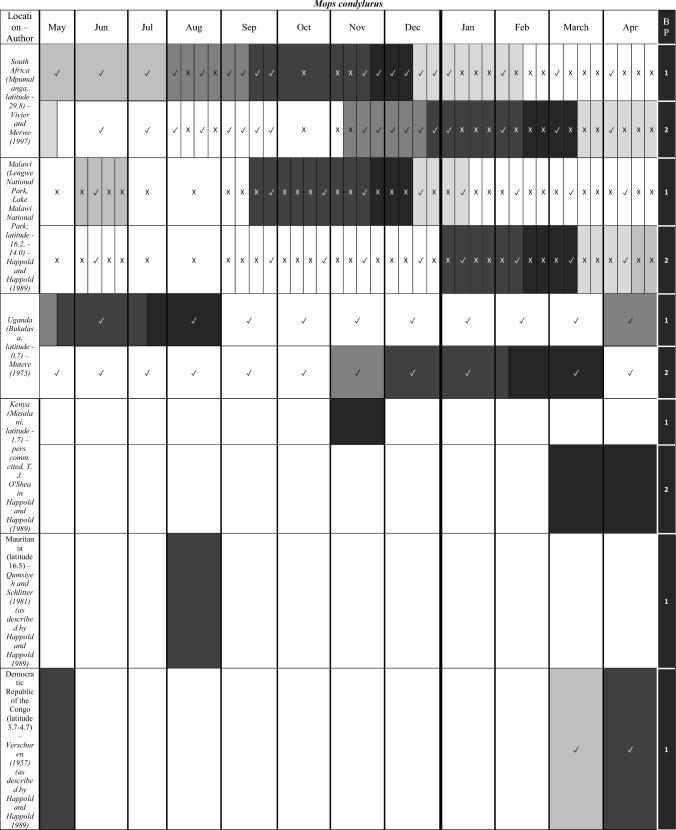

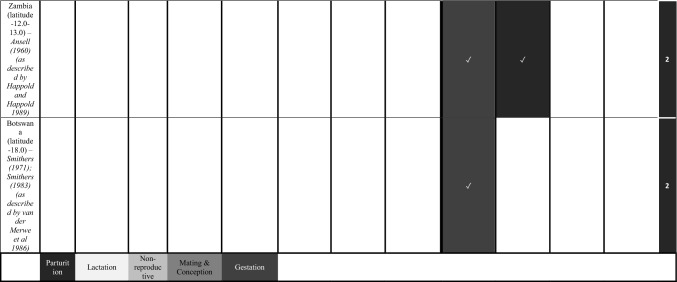
Female Reproductive Cycle of *M. condylurus*.

Width of cell represents temporal resolution of data (week, two-week, or month). X indicates weeks/months bats were not captured, ✓ where bats were captured, and blank cells where capture dates where not reported. Colored cells represent reproduction stage. Colored cells where no bats were captured indicate extrapolation from available data. White cells show where no reproduction information was provided. Rows per study are separated to show the timing of reproductive events per reported birth pulse (BP).

**Table 2:**
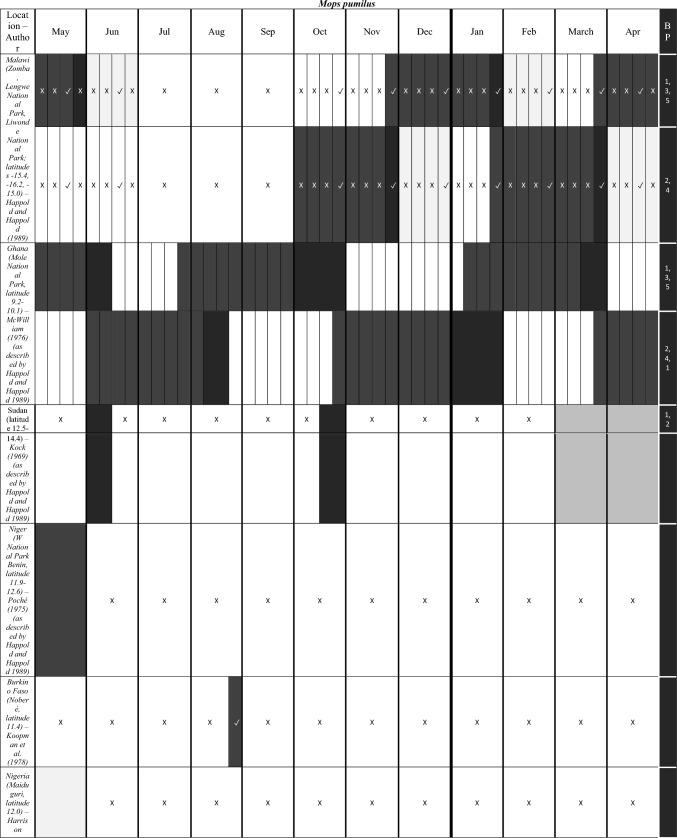

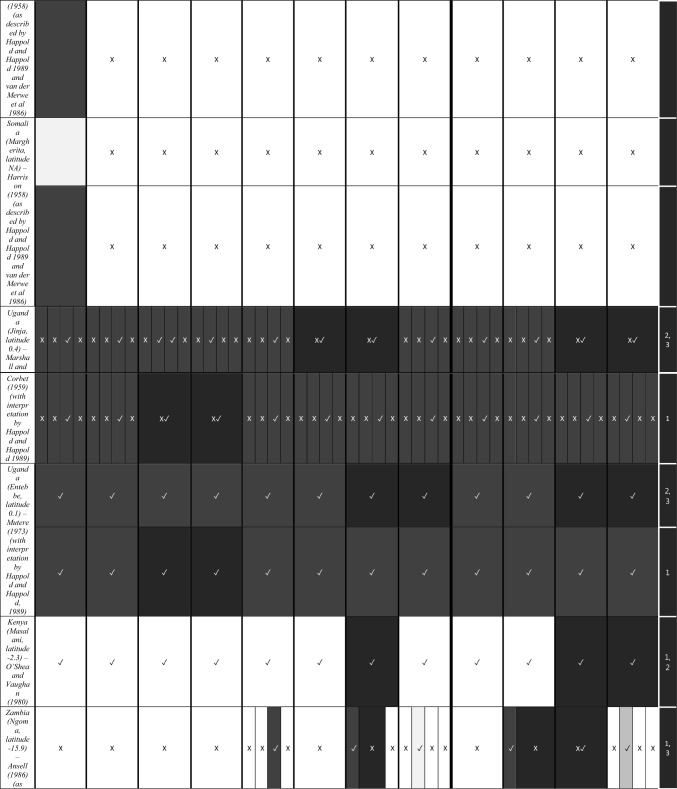

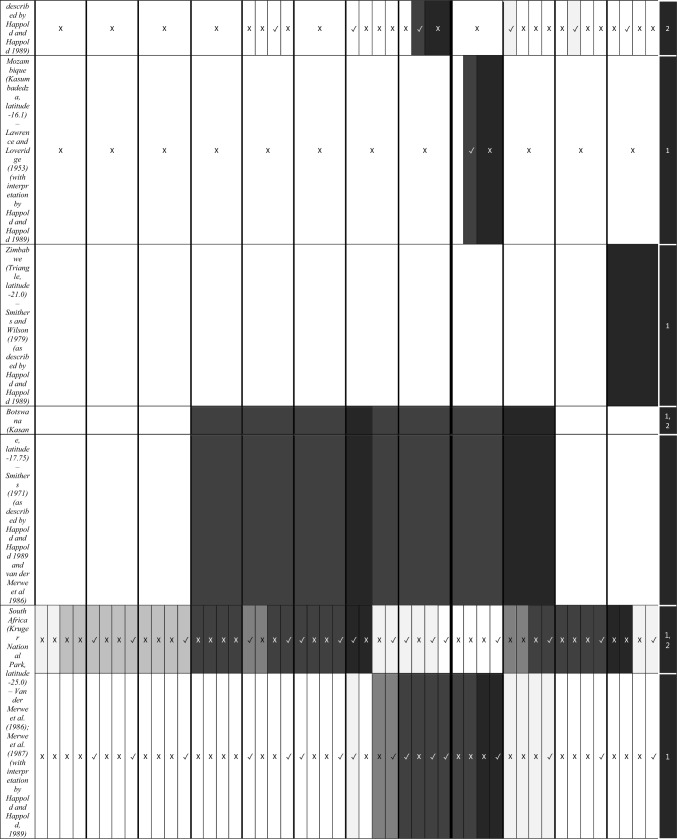

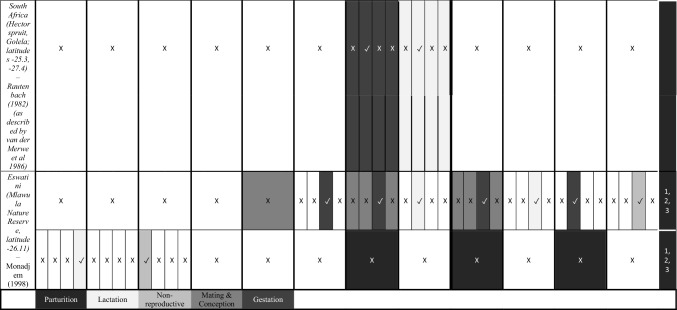
Female Reproductive Cycle of *M. pumilus*.

As in Table 1, width of cell represents temporal resolution of data (week, two-week, or month). X indicates weeks/months bats were not captured, ✓ where bats were captured, and blank cells where capture dates where not reported. Colored cells represent reproduction stage. Colored cells where no bats were captured indicate extrapolation from available data. White cells show where no reproduction information was provided. Rows per study are separated to show the timing of reproductive events per reported birth pulse (BP).

For both species, the lower synchrony in birth pulses should increase the probability of long-term viral maintenance within these populations, when compared with expectations from previous estimates, and with model bat species used in the disease literature (Pteropodid species with higher birth synchronicity, ~ 3–4 weeks) (Amman et al. 2012; Peel et al. 2014). For *M. condylurus*, our estimate of birth pulse synchronicity was considerably longer than previous “best-guess” estimates (~ 8.5 weeks, compared to 3–4 weeks as in Mutere 1973; O’Shea and Vaughan 1980; Happold and Happold 1989; and Vivier and Merwe 1997), while for *M. pumilus*, reproductive stages were wide and largely non-defined, and gestation spanned the entire study period (12 weeks). This was supported by the asynchrony observed in gestational stage and reproductive class per week of the study—e.g., females were observed to be a mix of early-, mid- and late-stage gestation at any given time during the birth pulse. The absence of a discernible birth pulse for *M. pumilus* may be consistent with a pattern of continuous breeding, as described at other equatorial locations for this species (e.g., Uganda) (Marshall and Corbet 1959; Mutere 1973; Happold and Happold 1989) or potentially reflect a mix of two, tight birth pulses in November and March–April (O’Shea and Vaughan 1980). Lower synchrony in birth pulses should facilitate viral maintenance by extending the timing of susceptible individual influx into the population, thereby decreasing the likelihood of stochastic pathogen extinction (Hayman 2015).

We observed a relatively balanced male-to-female population composition for both species (1:1.08 for *M. condylurus*, and 1:1.84 for *M. pumilus*), which will underlie the maximum potential magnitude of their respective birth pulses. Higher relative abundances of females will increase the maximum number of susceptible juveniles added to the population per birth pulse, and increase the proportion of contacts between susceptible and infected individuals, ultimately increasing the rate of transmission during and following the birthing period (Peel et al. 2014; Plowright et al. 2016; Baker et al. 2020). For *M. condylurus*, we observed proportionally more females than expected based on previous estimates of male-to-female ratios (2.6:1, as reported in Happold and Happold (1989)), which should increase the maximum expected magnitude of the birth pulse. For *M. pumilus* this was proportionally less, (previous reports up to 21 females per male) (McWilliam 1988; Bouchard 2001), which should decrease the maximum expected magnitude of the birth pulse. As aforementioned, these species do not form maternity roosts; the sex ratio reported here reflects the sex ratio of the broader population sampled.

The magnitude of the birth pulse will also be driven by the proportion of reproductively active females; a population with proportionally more females will have greater potential to produce more susceptible juveniles, but only if reproductively active. We observed a high proportion of reproduction in each species (~ 85 and 90% for *M. condylurus* and *M. pumilus* respectively). This was proportionally fewer than expected for *M. condylurus* (previously reported at 100%), but as expected for *M. pumilus* in this location. The proportion of reproductively active females will likely vary year-to-year for each species with environmental conditions (e.g., rainfall and food availability), and will likely vary widely by location for *M. pumilus*; for example, 93% of females have been observed to birth to 3 litters in Ghana, and 80% in Malawi. The proportion of females achieving the maximum of 5 births/year has not been determined (Marshall and Corbet 1959; Mutere 1973; McWilliam 1976; Happold and Happold 1989). Potential variability should be considered in spatial–temporal models of pathogen risk.

Birthing events of African molossid bats have been linked to human Ebola virus disease spillover previously (Hranac et al. 2019), supporting the plausibility that seasonal birthing drives epidemic cycles in these species. The specific effects of birth pulse magnitude and timing on infection dynamics are more difficult to predict from existing modeling work (Peel et al. 2014; Hayman 2015). The intervals between birthing pulses are uneven for *M. condylurus* (seven and three months) (pers. comm. by T. J. O'Shea cited in Happold and Happold (1989)). This contrasts to even intervals used in prior models of bi-annual birthing in Pteropodids and may have counterintuitive effects on pathogen dynamics (Hayman 2015). In addition, the potential magnitude of the birth pulse will be driven (in part) by the male-to-female ratio observed in the population, the proportion of females reproducing per birth pulse, population size, and the maturation period, which are ecological features of *M. condylurus* that differ from generic Pteropodid bat parameters used in Hayman (2015). Species-specific models are needed to interpret how, specifically, identified attributes of the birth pulse may interact with other features of *M. condylurus*-ebolavirus ecology to influence infection dynamics.

We acknowledge the difficulties in obtaining accurate estimates on birth pulse duration and synchronicity. We defined the beginning of the birth pulse by the first observation of females lactating but not gestating, and we observed three *M. condylurus* in this category during the first week. While these were few, and this did not significantly deviate from observations of lactating and pregnant females, it does indicate that at least some females had birthed by the start of the study, suggesting that 8.5 weeks could be an underestimate of the pulse duration. On the other hand, we cannot determine whether lactating non-pregnant females caught early in the birth pulse (~ January/February) were females that had given birth in the recorded birth pulse, as interpreted, or had given birth in the prior birth pulse (~ November, Happold and Happold (1989)). This is plausible, given a lactation period of ~ 50–60 days (Vivier and Merwe 1997). If the latter is true, our estimate of synchronicity for *M. condylurus* could be lower than 8.5 weeks. Regardless, the co-occurrence of disparate gestational stages within weekly timepoints suggests a level of asynchrony in mating and timing of birth for this species. In addition, an even wider span of gestational stages has been observed at this location during this timeframe, from previous capture events by the authors (Appendix S5). 10, 21 and 56 *M. condylurus* at early-, mid-, and late-stage gestation stages, respectively (here, directly determined by dissection and examination of fetus size) were captured over a 7-day capture period in early March 2019 (Appendix S5). As the gestation period for *M. condylurus* is 85–90 days (~ 3 months), this range of gestation stages (particularly those observed within a one-week period in 2019) implies an equally wide period of mating and birthing. These data collectively support a long birth pulse, as interpreted, and the effects on viral dynamics from low synchrony in the birth pulse remain as predicted.

In addition, if females at early gestation were miscategorized as non-reproductive, it is possible that the true proportion of non-reproductive females is lower than the estimated 16.6%. The observation of early gestation by dissection of adults in late March 2019 underscores this possibility (Appendix S5). However, we would note that non-reproductive adult females were observed throughout the study period, including April, when gestation and/or lactation should be obvious. As such, it is reasonable to conclude that less than 100% of females were pregnant immediately prior to the birth pulse, which is less than previous estimates (Mutere 1973; Happold and Happold 1989; Vivier and Merwe 1997).

## Conclusion

In summary, we provide the first estimates of synchronicity, magnitude, and timing of seasonal birthing in a population of *M. condylurus* and *M. pumilus*, underpinned by high-resolution temporal data in a region of zoonotic significance. We show that population-level synchronicity of the birth pulse in *M. condylurus* is longer than previously estimated (~ 8.5 vs 3–4 weeks). This was even wider and less defined for *M. pumilus*. This is predicted to increase the likelihood of filovirus persistence under conditions of bi-annual birthing. We also observed proportionally more female *M. condylurus*, and less female *M. pumilus,* than expected based on previous estimates of male-to-female ratios, but proportionally fewer reproductively active females. These observations will have countering effects on the potential magnitude of the birth pulse (a population with proportionally more females will have greater potential to produce more susceptible juveniles, but only if reproductively active). Species-specific models are needed to interpret how, specifically, these identified attributes of the birth pulse may interact with other features of molossid ebolavirus ecology to influence infection dynamics. Basic ecological data, such as these presented, are fundamental to research efforts toward understanding the dynamics and drivers of infection in wildlife reservoir hosts and will be imperative to future efforts of epidemic prevention and preparedness.

### Supplementary Information

Below is the link to the electronic supplementary material.

Supplementary file1 (DOCX 1837 kb)
