# Supplementary material for: Kenyan Free-Tailed Bats Demonstrate Seasonal Birth Pulse Asynchrony with Implications for Virus Maintenance
Source: Ecohealth. 2024 Feb 19;21(1):94–111. doi: 10.1007/s10393-024-01674-x (PMC11127837; doi:10.1007/s10393-024-01674-x)
Supplement: Supplementary file 1 — Supplementary file1 (DOCX 1837 kb) [file 10393_2024_1674_MOESM1_ESM.docx]

**Supplementary Information**

The following supplementary information is available for this article online

**Appendix S1-S5**

**Seasonal births in Kenyan free-tailed bats: within-pulse asynchrony and virus maintenance**

Tamika J. Lunn*, Reilly T. Jackson, Paul W. Webala, Joseph Ogola, Kristian M. Forbes

*Author for correspondence:

Tamika J. Lunn

Email: tjlunn@uark.edu

Appendix S1: Supporting materials and methods


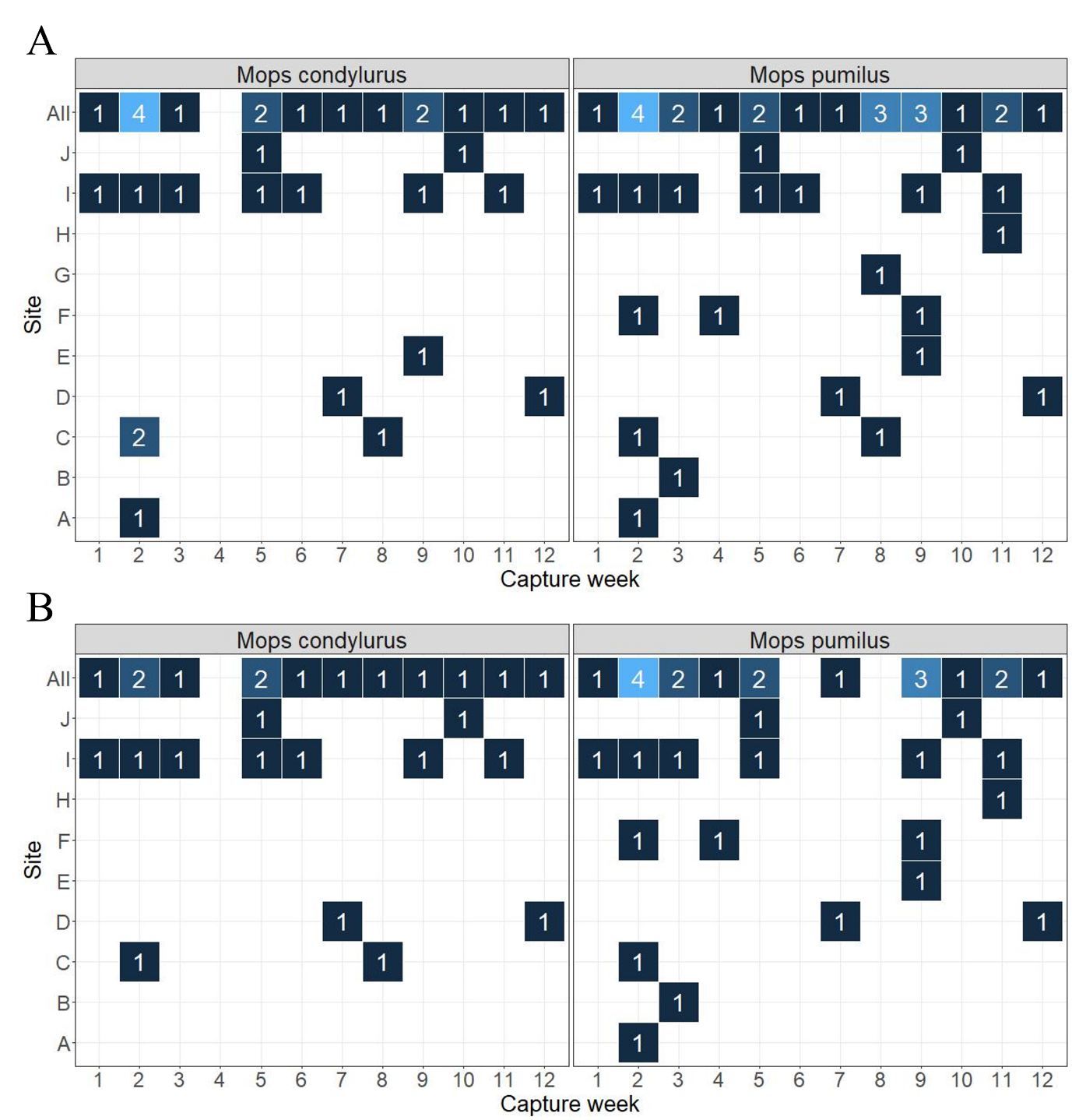


Figure S1: Tally of capture nights per week, per site, across the study period. A shows all capture nights, B shows capture nights where at least one adult female was caught. Weeks span the last week in January to the second week of April 2022.

Appendix S2: Sensitivity analyses

Table S1: Data on reproductive states and results of chi-square test for captured bats. Chi-square comparisons were made per week and included: 1) numbers of gestating vs non-gestating females; 2) numbers of females at early vs mid vs late gestational stages; 3) numbers of females that were lactating and gestating simultaneously, vs those lactating but not gestating, vs those that were post-lactating. NA is noted for weeks where no females were captured (and therefore, no females in categories). P-values were generated using Monte Carlo simulation of p-values for chi-squared tests. P-values below 0.05 are bolded. Red text highlights cases where the significance level deviates from results presented in the main text, where results either became significant (*M. condylurus* week 1), or lost significance (*M. condylurus* week 6, *M. pumilus* weeks 3 and 7) as a result of removing bats captured more than once from the datasets. There is no repetition of individuals within weeks or across weeks.

|  |  |  | | | | **1** | | | | | **2** | | | | | | | | **3** | | | | | | |  |
| --- | --- | --- | --- | --- | --- | --- | --- | --- | --- | --- | --- | --- | --- | --- | --- | --- | --- | --- | --- | --- | --- | --- | --- | --- | --- | --- |
| **Wk** | **Date range** | **Total** | | | | **Gestat.** | | **Not gestat.** | **χ^2^** | **p** | **Early-gestat.** | | **Mid-gestat.** | **Late-gestat.** | **χ^2^** | | **p** | | **Lactating & not gestat.** | **Lactating & gestat. (B)** | **Post- lactating** | **χ^2^** | | **p** | | **Non-repro** |
| *Mops condylurus* | | | | | | | | | | | | | | | | | | | | | | | | | | |
| 1 | 24th – 30th Jan | | 13 | | | **2** | | **11** | **6.23** | **0.0267** | 1 | | 0 | 1 | 1.00 | | 1.0000 | | 3 | 1 | 5 | 2.67 | | 0.3218 | | 4 |
| 2 | 31st Jan – 6th Feb | | 22 | | | **17** | | **5** | **6.55** | **0.0178** | **2** | | **2** | **13** | **14.24** | | **0.001** | | **0** | **10** | **3** | **12.15** | | **0.0020** | | 3 |
| 3 | 7th – 13th Feb | | 5 | | | 5 | | 0 | 5.00 | 0.0619 | 0 | | 1 | 4 | 5.20 | | 0.1386 | | 0 | 2 | 1 | 2.00 | | 0.7731 | | 0 |
| 4 | 14th – 20th Feb | | 0 | | | NA | | NA | NA | NA | NA | | NA | NA | NA | | NA | | NA | NA | NA | NA | | NA | | 0 |
| 5 | 21st –27th Feb | | 26 | | | **20** | | **6** | **7.54** | **0.0100** | **0** | | **5** | **15** | **17.5** | | **0.0003** | | **3** | **14** | **3** | **12.10** | | **0.0024** | | 3 |
| 6 | 28th Feb – 6th Mar | | 13 | | | 8 | | 5 | 0.69 | 0.5756 | 0 | | 3 | 5 | 4.75 | | 0.1086 | | **3** | **7** | **0** | **7.40** | | **0.0243** | | 2 |
| 7 | 7th – 13th Mar | | 12 | | | 8 | | 4 | 1.33 | 0.3916 | **0** | | **6** | **2** | **7.00** | | **0.0303** | | 2 | 7 | 2 | 4.55 | | 0.1314 | | 1 |
| 8 | 14th – 20th Mar | | 11 | | | 8 | | 3 | 2.27 | 0.2261 | **0** | | **1** | **7** | **10.75** | | **0.0067** | | **0** | **6** | **0** | **12.00** | | **0.0040** | | 3 |
| 9 | 21st – 27th Mar | | 9 | | | 2 | | 7 | 2.78 | 0.1715 | 0 | | 0 | 2 | 4.00 | | 0.3394 | | **6** | **2** | **0** | **7.00** | | **0.0345** | | 1 |
| 10 | 28th Mar – 3rd Apr | | 39 | | | **1** | | **38** | **35.10** | **0.0001** | 0 | | 0 | 1 | 2.00 | | 1.0000 | | **33** | **1** | **0** | **62.18** | | **0.0001** | | 5 |
| 11 | 4th – 10th Apr | | 7 | | | **0** | | **7** | **7.00** | **0.0141** | 0 | | 0 | 0 | NA | | NA | | 2 | 0 | 0 | 4.00 | | 0.3282 | | 5 |
| 12 | 11th – 17th Apr | | 9 | | | **0** | | **9** | **9.00** | **0.0040** | 0 | | 0 | 0 | 1.00 | | 1.0000 | | **8** | **0** | **1** | **12.67** | | **0.0027** | | 0 |
| *Mops pumilus* | | | | | | | | | | | | | | | | | | | | | | | | | | |
| 1 | 24th – 30th Jan | | 5 | | | 2 | | 3 | 0.20 | 1.0000 | 1 | | 0 | 1 | 1.00 | | 1.0000 | | 0 | 0 | 2 | 4.00 | | 0.3388 | | 2 |
| 2 | 31st Jan – 6th Feb | | 35 | | | **34** | | **1** | **31.11** | **0.0001** | **0** | | **15** | **18** | **16.91** | | **0.0004** | | **1** | **28** | **2** | **45.35** | | **0.0001** | | 0 |
| 3 | 7th – 13th Feb | | 41 | | | **36** | | **5** | **23.44** | **0.0001** | 6 | | 18 | 12 | 6.00 | | 0.0602 | | **5** | **30** | **5** | **31.25** | | **0.0001** | | 0 |
| 4 | 14th – 20th Feb | | 6 | | | 5 | | 1 | 2.67 | 0.2272 | 1 | | 3 | 1 | 1.60 | | 0.618 | | 1 | 5 | 0 | 7.00 | | 0.0538 | | 0 |
| 5 | 21st –27th Feb | | 3 | | | 2 | | 1 | 0.33 | 1.0000 | 0 | | 1 | 1 | 1.00 | | 1.0000 | | 1 | 1 | 0 | 1.00 | | 1 | | 0 |
| 6 | 28th Feb – 6th Mar | | 0 | | | NA | | NA | NA | NA | NA | | NA | NA | NA | | NA | | NA | NA | NA | NA | | NA | | 0 |
| 7 | 7th – 13th Mar | | 17 | | | **2** | | **15** | **9.94** | **0.0023** | 0 | | 1 | 1 | 1.00 | | 1.0000 | | 6 | 1 | 1 | 6.25 | | 0.0588 | | 9 |
| 8 | 14th – 20th Mar | | 0 | | | NA | | NA | NA | NA | NA | | NA | NA | NA | | NA | | NA | NA | NA | NA | | NA | | 0 |
| 9 | 21st – 27th Mar | | 76 | | | 32 | | 44 | 1.90 | 0.2019 | **0** | | **24** | **8** | **28.00** | | **0.0001** | | **36** | **14** | **6** | **25.86** | | **0.0001** | | 7 |
| 10 | 28th Mar – 3rd Apr | | 1 | | | 0 | | 1 | 1.00 | 1.0000 | 0 | | 0 | 0 | NA | | NA | | 1 | 0 | 0 | 2.00 | | 1.0000 | | 0 |
| 11 | 4th – 10th Apr | | 41 | | | 27 | | 14 | 4.12 | 0.0577 | **0** | | **17** | **10** | **16.22** | | **0.0002** | | 10 | 14 | 10 | 0.94 | | 0.6411 | | 4 |
| 12 | 11th – 17th Apr | | 38 | | | 16 | | 22 | 0.95 | 0.4146 | **0** | | **6** | **10** | **9.50** | | **0.0069** | | 17 | 9 | 7 | 5.09 | | 0.0866 | | 4 |
|  |  |  | | ***Additional breakdown of gestation stage for lactating females (B)*** | | | | | | | | | | | | | | | | | | | | | | |
| Species | | | | |  | | Lactating during early gestation | | | | | Lactating during mid gestation | | | |  | | Lactating during late gestation | | | | |  | | Lactating, not gestating | |
| *Mops condylurus* | | | | |  | | 1 | | | | | 14 | | | |  | | 35 | | | | |  | | 60 | |
| *Mops pumilus* | | | | |  | | 6 | | | | | 57 | | | |  | | 39 | | | | |  | | 78 | |

Appendix S3: Supporting results


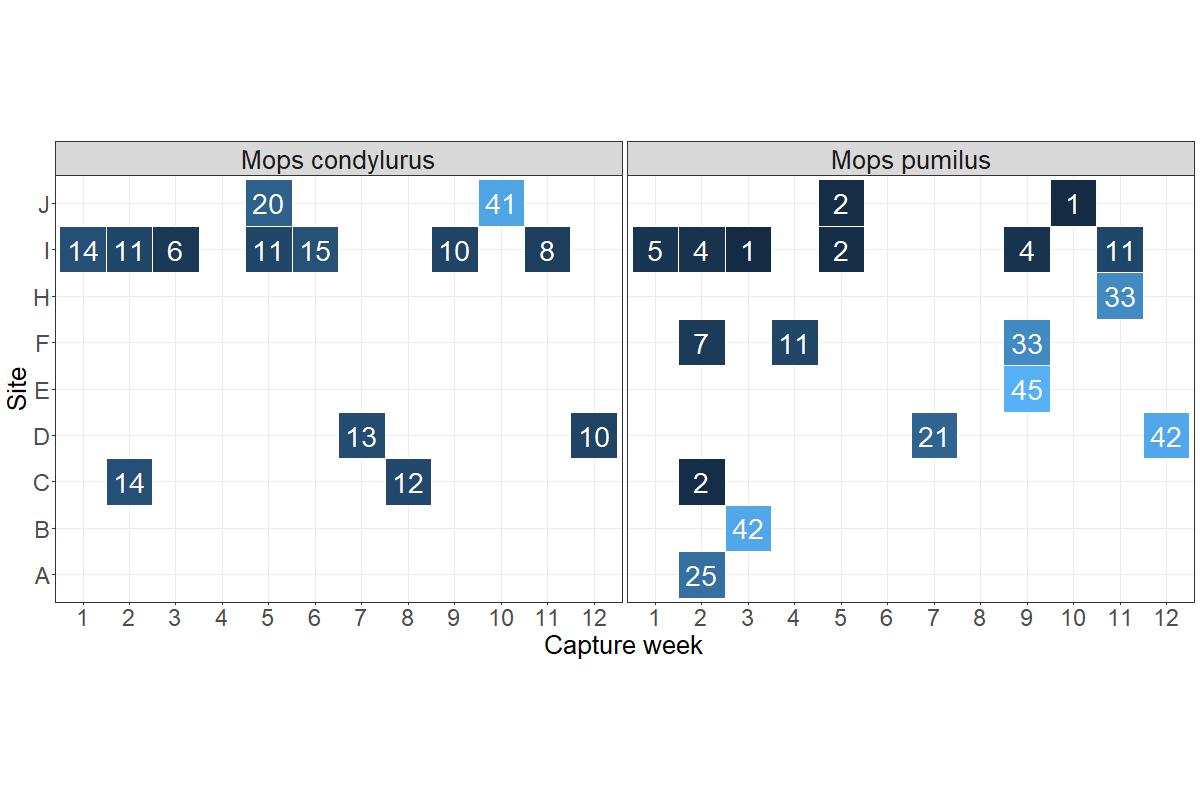


Figure S2: Number of adult females captured per week, per site, across the study period. Weeks span the last week in January to the second week of April 2022.

Table S2: Demographic breakdown of captured bats. Numbers outside of parentheses show individual bats captured, and numbers inside of parentheses show total capture numbers including recaptures.

| **Adult female** | **Adult male** | **Juvenile female** | **Juvenile male** |
| --- | --- | --- | --- |
| *Mops condylurus* | | | |
| 175 (185) | 152 (177) | 29 (30) | 39 (39) |
| Mops pumilus | | | |
| 278 (291) | 118 (126) | 33 (33) | 51 (54) |


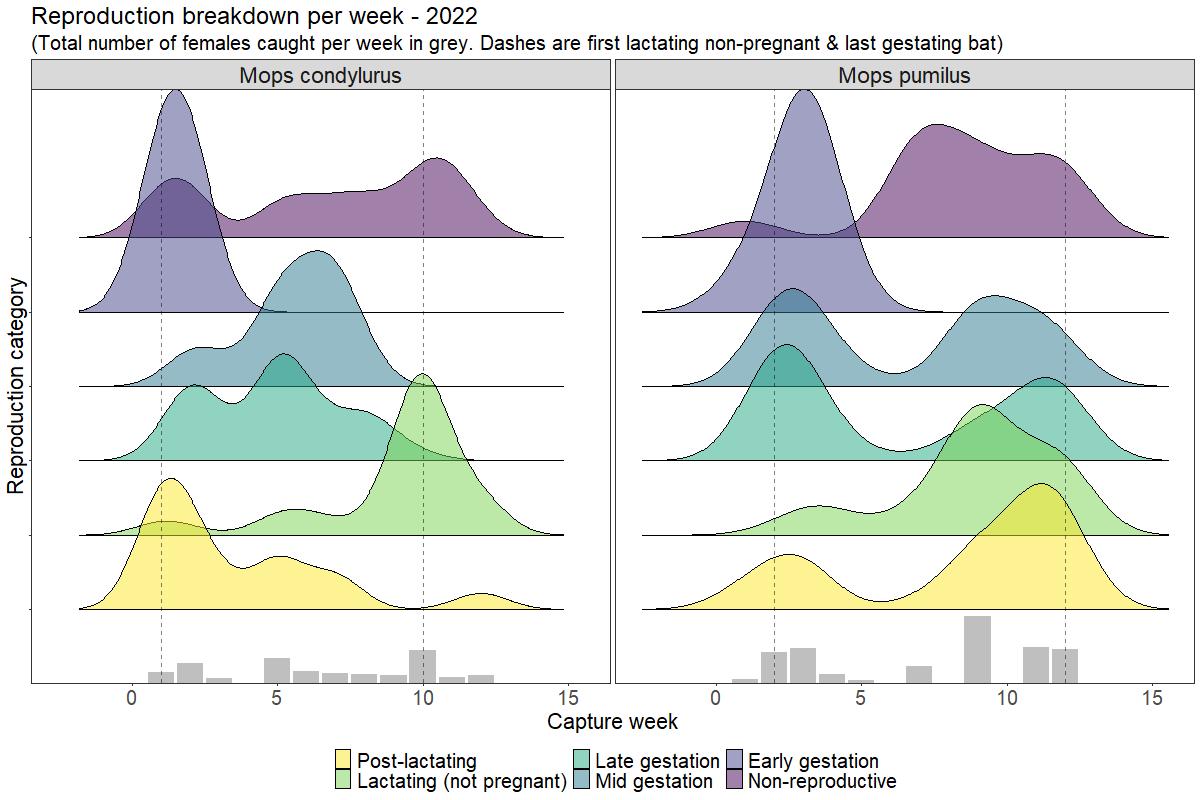


Figure S3: Further information on gestational stage over the study period. Breakdown of female *Mops condylurus* reproductive states across a single birth pulse in south-eastern Kenya. Density plots show the relative frequency of bats captured at each reproductive state. Weeks span the last week in January to the second week of April 2022: Week 1) 24th – 30th Jan; Week 2) 31st Jan – 6th Feb; Week 3) 7th – 13th Feb; Week 4) 14th – 20th Feb; Week 5) 21st –27th Feb; Week 6) 28th Feb – 6th Mar; Week 7) 7th – 13th Mar; Week 8) 14th – 20th Mar; Week 9) 21st – 27th Mar; Week 10) 28th Mar – 3rd Apr; Week 11) 4th – 10th Apr; Week 12) 11th – 17th Apr. The total number of female bats caught per week are shown by the grey bars. Dashes indicate the first lactating non-pregnant (left) and last gestating (right) bat captured.

Table S3: Data on reproductive states and results of chi-square test for captured bats. Chi-square comparisons were made per week and included: 1) numbers of gestating vs non-gestating females; 2) numbers of females at early vs mid vs late gestational stages; 3) numbers of females that were lactating and gestating simultaneously, vs those lactating but not gestating, vs those that were post-lactating. NA is noted for weeks where no females were captured (and therefore, no females in categories). P-values were generated using Monte Carlo simulation of p-values for chi-squared tests. P-values below 0.05 are bolded. There is no repetition of individuals within weeks (within single Chi-square tests), but there is repetition of individuals across weeks (across independent tests).

|  |  |  | **1** | | | | **2** | | | | | | | | **3** | | | | | |  | |
| --- | --- | --- | --- | --- | --- | --- | --- | --- | --- | --- | --- | --- | --- | --- | --- | --- | --- | --- | --- | --- | --- | --- |
| **Wk** | **Date range** | **Total** | **Gestat.** | **Not gestat.** | **χ^2^** | **p** | **Early-gestat.** | | **Mid-gestat.** | **Late-gestat.** | **χ^2^** | | **p** | | **Lactating & not gestat.** | **Lactating & gestat. (B)** | **Post- lactating** | | **χ^2^** | | **p** | **Non-repro** |
|  |  |  | *Mops condylurus* | | | | | | | | | | | | | | | | | | | |
| 1 | 24th – 30th Jan | 14 | 3 | 11 | 4.57 | 0.0556 | 2 | | 0 | 1 | 2.00 | | 0.7765 | | 3 | 1 | 6 | | 3.80 | | 0.1742 | 4 |
| 2 | 31st Jan – 6th Feb | 25 | **18** | **7** | **4.84** | **0.0420** | **2** | | **2** | **14** | **16.00** | | **0.0005** | | **1** | **11** | **3** | | **11.20** | | **0.0032** | 4 |
| 3 | 7th – 13th Feb | 6 | 5 | 1 | 2.67 | 0.2224 | 0 | | 1 | 4 | 5.20 | | 0.1382 | | 0 | 2 | 1 | | 2.00 | | 0.7729 | 0 |
| 4 | 14th – 20th Feb | 0 | NA | NA | NA | NA | NA | | NA | NA | NA | | NA | | NA | NA | NA | | NA | | NA | 0 |
| 5 | 21st –27th Feb | 31 | **24** | **7** | **9.32** | **0.0038** | **0** | | **5** | **19** | **24.25** | | **0.0001** | | **4** | **18** | **3** | | **16.88** | | **0.0005** | 3 |
| 6 | 28th Feb – 6th Mar | 15 | 10 | 5 | 1.67 | 0.3036 | **0** | | **3** | **7** | **7.40** | | **0.0222** | | **3** | **9** | **0** | | **10.50** | | **0.0035** | 2 |
| 7 | 7th – 13th Mar | 13 | 8 | 5 | 0.69 | 0.5834 | **0** | | **6** | **2** | **7.00** | | **0.0341** | | 2 | 7 | 2 | | 4.55 | | 0.1337 | 2 |
| 8 | 14th – 20th Mar | 12 | 9 | 3 | 3.00 | 0.1452 | **0** | | **1** | **8** | **12.67** | | **0.0028** | | **0** | **7** | **0** | | **14.00** | | **0.0013** | 3 |
| 9 | 21st – 27th Mar | 10 | 2 | 8 | 3.60 | 0.113 | 0 | | 0 | 2 | 4.00 | | 0.3396 | | **7** | **2** | **0** | | **8.67** | | **0.0165** | 1 |
| 10 | 28th Mar – 3rd Apr | 41 | **1** | **40** | **37.10** | **0.0001** | 0 | | 0 | 1 | 2.00 | | 1.0000 | | **35** | **1** | **0** | | **66.17** | | **0.0001** | 5 |
| 11 | 4th – 10th Apr | 8 | **0** | **8** | **8.00** | **0.0084** | 0 | | 0 | 0 | NA | | NA | | 3 | 0 | 0 | | 6.00 | | 0.1098 | 5 |
| 12 | 11th – 17th Apr | 10 | **0** | **10** | **10.00** | **0.0019** | 0 | | 0 | 0 | 2.99 | | 0.7765 | | **8** | **0** | **1** | | **12.67** | | **0.0025** | 1 |
|  | Total | 185 | 80 | 105 |  |  | 4 | | 18 | 58 |  | |  | | 66 | 58 | 16 | |  | |  | 30 |
|  |  |  | *Mops pumilus* | | | | | | | | | | | | | | | | | | | |
| 1 | 24th – 30th Jan | 5 | 2 | 3 | 0.20 | 1 | 1 | | 0 | 1 | 1.00 | | 1.0000 | | 0 | 0 | 2 | | 4.00 | | 0.3417 | 2 |
| 2 | 31st Jan – 6th Feb | 38 | **37** | **1** | **34.11** | **0.0001** | **0** | | **17** | **19** | **18.17** | | **0.0001** | | **1** | **30** | **2** | | **49.27** | | **0.0001** | 0 |
| 3 | 7th – 13th Feb | 43 | **38** | **5** | **25.33** | **0.0001** | **6** | | **19** | **13** | **6.684** | | **0.0359** | | **5** | **32** | **5** | | **34.71** | | **0.0001** | 0 |
| 4 | 14th – 20th Feb | 11 | 6 | 5 | 0.09 | 1 | 1 | | 3 | 2 | 1.00 | | 0.8735 | | 5 | 6 | 0 | | 5.636 | | 0.0552 | 0 |
| 5 | 21st –27th Feb | 4 | 3 | 1 | 1.00 | 0.6311 | 0 | | 2 | 1 | 2.00 | | 0.7799 | | 1 | 1 | 0 | | 1 | | 1.0000 | 0 |
| 6 | 28th Feb – 6th Mar | 0 | NA | NA | NA | NA | NA | | NA | NA | NA | | NA | | NA | NA | NA | | NA | | NA | 0 |
| 7 | 7th – 13th Mar | 21 | **2** | **19** | **13.76** | **0.0002** | 0 | | 1 | 1 | 1.00 | | 1.0000 | | **8** | **1** | **1** | | **9.80** | | **0.0123** | 11 |
| 8 | 14th – 20th Mar | 0 | NA | NA | NA | NA | NA | | NA | NA | NA | | NA | | NA | NA | NA | | NA | | NA | 0 |
| 9 | 21st – 27th Mar | 82 | 34 | 48 | 2.39 | 0.1504 | **0** | | **26** | **8** | **31.29** | | **0.0001** | | **39** | **15** | **7** | | **27.28** | | **0.0001** | 8 |
| 10 | 28th Mar – 3rd Apr | 1 | 0 | 1 | 1.00 | 1 | 0 | | 0 | 0 | NA | | NA | | 1 | 0 | 0 | | 2.00 | | 1.0000 | 0 |
| 11 | 4th – 10th Apr | 44 | **29** | **15** | **4.46** | **0.0487** | **0** | | **17** | **12** | **15.79** | | **0.0006** | | 11 | 16 | 10 | | 1.68 | | 0.4473 | 4 |
| 12 | 11th – 17th Apr | 42 | 17 | 25 | 1.52 | 0.2765 | **0** | | **6** | **11** | **10.71** | | **0.0067** | | 18 | 10 | 7 | | 5.54 | | 0.0671 | 6 |
|  | Total | 291 | 168 | 123 |  |  | 8 | | 91 | 68 |  | |  | | 89 | 111 | 34 | |  | |  | 31 |
|  |  |  | ***Additional breakdown of gestation stage for lactating females (B)*** | | | | | | | | | | | | | | | | | | | |
| Species | | |  | Lactating during early gestation | | | | Lactating during mid gestation | | | |  | | Lactating during late gestation | | | |  | | Lactating, not gestating | | |
| *Mops condylurus* | | |  | 1 | | | | 14 | | | |  | | 43 | | | |  | | 66 | | |
| *Mops pumilus* | | |  | 6 | | | | 60 | | | |  | | 45 | | | |  | | 89 | | |

Appendix S4: Recapture data

4.6% of adult, female *M. condylurus* (8 of 175 individuals) and 4.7% of adult, female *M. pumilus* (13 of 278 individuals) were recaptured more than once. Of the 8 recaptured *M. condylurus* individuals, 7 were captured twice each (i.e., initial capture and banding, with one subsequent recapture), and one was captured four times. All 13 recaptured *M. pumilus* individuals were captured twice each.

Three of each species were recaptured within the gestational period, and of these, only two transitioned between gestational classes (mid to late gestation, both *M. pumilus*) (Figure S4). The maximum period between recapture within the gestational period, was 41 days for *M. condylurus* (with both captures occurring at late-stage gestation), and 66 days for *M. pumilus* (captures occurring at mid- and late-stage gestation). This information does not show how long gestational periods are, only that they are at least 41 and 66 days for *M. condylurus* and *M. pumilus*, but likely much longer.

Relating to timing of births, three *M. condylurus* transitioned between gestating and non-gestating, and five *M. pumilus* (Figure S5). Two *M. condylurus* birthed somewhere between weeks 5 and 9 (23rd February – 31st March), and one *M. condylurus* birthed somewhere between weeks 6 and 8 (3rd March – 21st March). *M. pumilus* birthed between weeks: 4 and 9 (18th February – 22nd March, one individual), 3 and 7 (7th February – 10th March, one individual), and 2 and 11 (31st January – 5th April, one individual). Conversely, two *M. pumilus* were lactating and subsequently became pregnant between weeks 4 and 9 (18th February – 22nd March).

Relating to duration of lactation, six *M. condylurus* were lactating across recaptures (five captured twice each, one captured four times), and nine *M. pumilus* (all captured twice each). The lactation window ranged 6-41 days for *M. condylurus*, and 31-64 days for *M. pumilus*. Again, this information does not show how long the lactation period is, only that it is at least this long for each of the species, but likely longer.


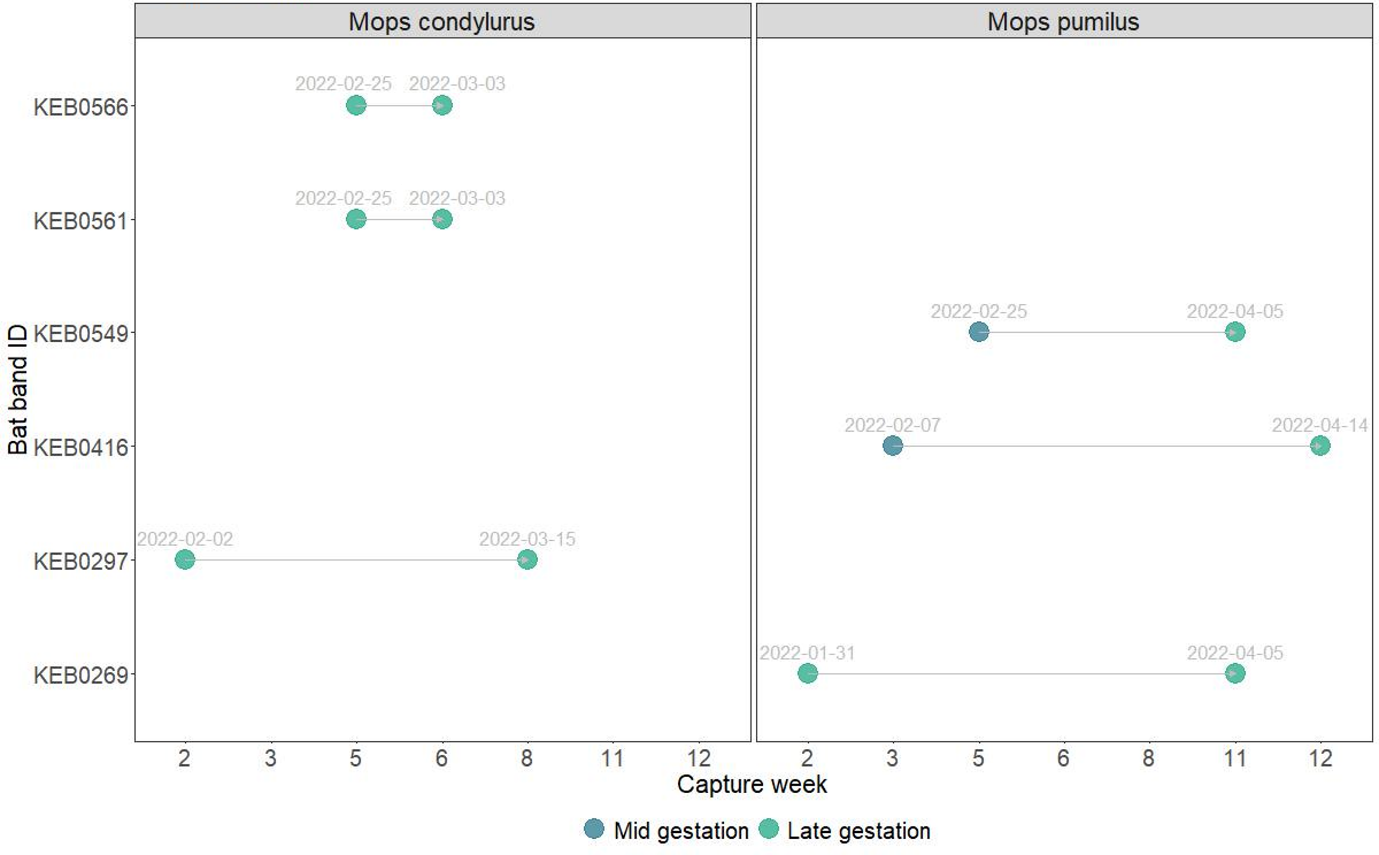


Figure S4: Bats recaptured within the gestational period.


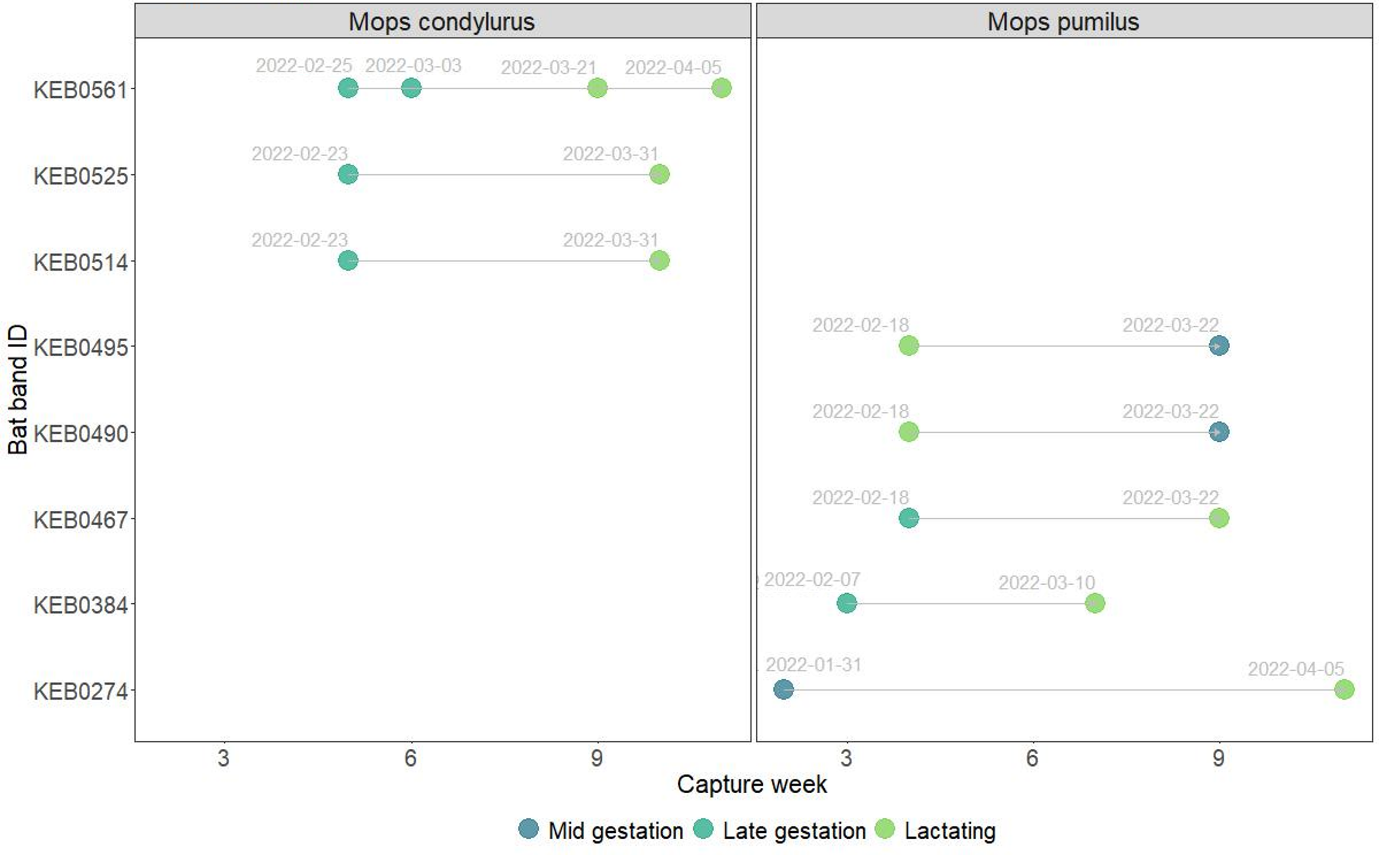


Figure S5: Timing of transition between gestation and non-gestation. Lactating bats (green) does not include bats simultaneously lactating and gestating.

Appendix S5: Unpublished data on gestation stage

*Mops condylurus* and *Mops pumilus* bats have been captured previously from the study area, for a concurrent study on Bombali virus in 2019 (Kareinen et al., 2019). The capture period spanned a one-week period in the same season, between March 2-10, 2019. Demographic information was collected as in the main text, but bats were euthanized and dissected with gestation confirmed by presence of a foetus. Stage of gestation was classified as early, mid, or late based on the approximate size of the foetus.

This tangential dataset includes 87 adult female *M. condylurus* and 26 adult female *M. pumilus*. Pregnant *M. condylurus* captured in this short timespan showed a wide range of gestational stages: 10, 21 and 56 *M. condylurus* at early-, mid-, and late-stage (Figure S2).


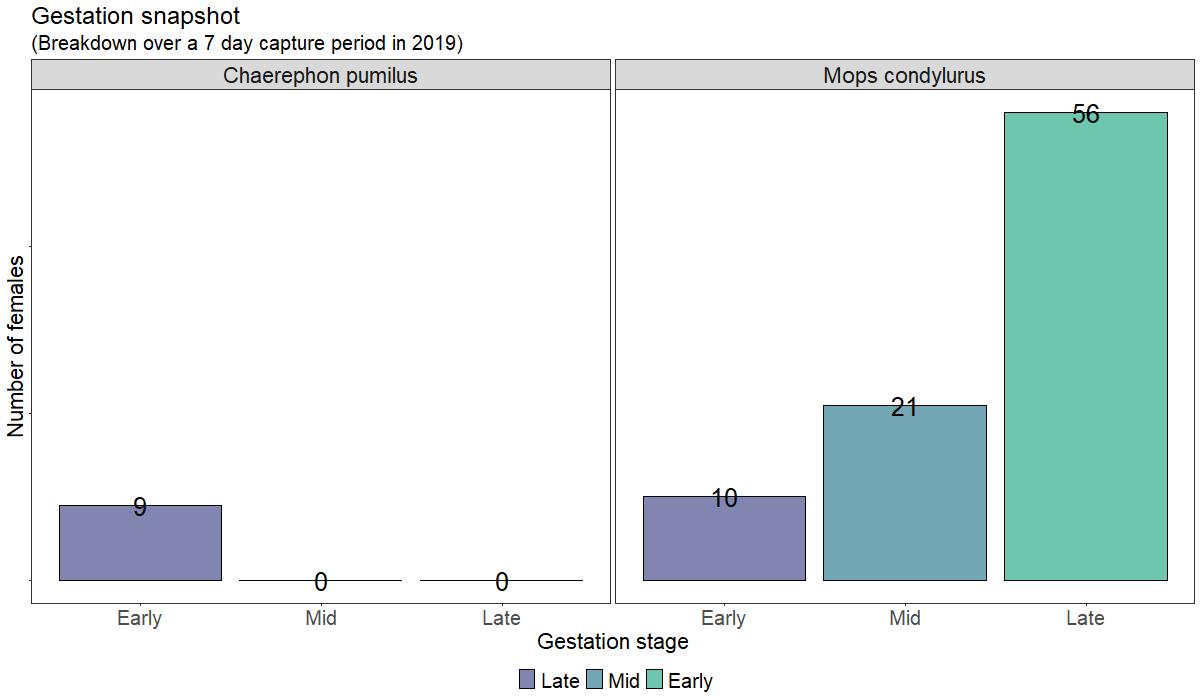


Figure S6: Snapshot of gestation class over a 7-day period (3^rd^ March – 10^th^ March 2019) determined by dissection and examination of foetus size.

**References**

Ansell WFH (1986) Some Chiroptera from south-central Africa

Ansell WFH (1960) Mammals of northern Rhodesia

Happold DCD, Happold M (1989) Reproduction of Angola free-tailed bats (*Tadarida condylura*) and little free-tailed bats (*Tadarida pumila*) in Malawi (Central Africa) and elsewhere in Africa. Journal of Reproduction and Fertility 85:133–149

Harrison DL (1958) A note on successive pregnancies in an African bat. Mammalia 22:592–595

Kock D (1969) Die Fledermaus-Fauna des Sudan:(Mammalia, Chiroptera). Mit 43 Tab. u. 20 Abb. Kramer

Koopman KF, Mumford RE, Heisterberg JF (1978) Bat records from Upper Volta, West Africa. American Museum novitates; no. 2643

Lawrence B, Loveridge A (1953) Zoological Results of a Fifth Expedition to East Africa: Mammals from Nyasaland and Tete. Museum of Comparative Zoology

Marshall AJ, Corbet PS (1959) The breeding biology of equatorial vertebrates: reproduction of the bat *Chaerephon hindei* Thomas at latitude 0° 26′ N. In: Proceedings of the Zoological Society of London. Wiley Online Library, pp 607–616

McWilliam AN (1976) The biology of *Tadarida (Chaerephon) pumila* (Cretzschmar) and partitioning of food resources among insectivorous bats in northern Ghana. B. Sc. Hons thesis, University of Aberdeen

Merwe MV der, Giddings SR, Rautenbach IL (1987) Post-partum oestrus in the little free-tailed bat, *Tadarida (Chaerephon) pumila* (Microchiroptera: Molossidae) at 24° S. Journal of Zoology 213:317–326

Monadjem A (1998) Reproduction in the little free-tailed bat Chaerephon pumila (Microchiroptera: Molossidae) in Swaziland. Durban Museum Novitates 23:39–41

Mutere FA (1973) Reproduction in two species of equatorial free‐tailed bats (Molossidae). African Journal of Ecology 11:271–280

O’Shea TJ, Vaughan TA (1980) Ecological observations on an East African bat community. Mammalia 44:485–496

Poché RM (1975) The bats of national park W, Niger, Africa

Qumsiyeh MB, Schlitter DA (1981) Bat records from Mauritania, Africa (Mammalia: Chiroptera). Carnegie Museum of Natural History

Rautenbach IL (1982) Mammals of the Transvaal Ecoplan Monograph No. 1. Colbyn Pretoria

Smithers RHN (1983) The mammals of the Southern African subregion. University of Pretoria

Smithers RHN (1971) The Mammals of Botswana. University of Pretoria

Smithers RHN, Wilson VJ (1979) Check list and atlas of the mammals of Zimbabwe Rhodesia. Trustees of the National Museums and Monuments Council of Zimbabwe Rhodesia 9:1–193

Van der Merwe M, Rautenbach IL, Van der Colf WJ (1986) Reproduction in females of the little free-tailed bat, *Tadarida (Chaerephon) pumila*, in the eastern Transvaal, South Africa. Reproduction 77:355–364

Verschuren J (1957) Écologie, biologie et systématique des cheiroptères. Institut des Parcs Nationaux du Congo Belge, Belgian Congo

Vivier L, Merwe M van der (1997) Reproduction in the female Angolan free‐tailed bat, *Tadarida (Mops) condylura* (Microchiroptera: Molossidae), in the eastern Transvaal, South Africa. Journal of Zoology 243:507–521
